# Blockade of Cellular Energy Metabolism through 6-Aminonicotinamide Reduces Proliferation of Non-Small Lung Cancer Cells by Inducing Endoplasmic Reticulum Stress

**DOI:** 10.3390/biology10111088

**Published:** 2021-10-22

**Authors:** Neha Kaushik, Nagendra Kumar Kaushik, Eun Ha Choi, June Hyun Kim

**Affiliations:** 1 Department of Biotechnology, College of Engineering, University of Suwon, Hwaseong 18323, Korea; jk8199@suwon.ac.kr; 2 Department of Electrical and Biological Physics, Plasma Bioscience Research Center & Applied Plasma Medicine Center, Kwangwoon University, Seoul 01897, Korea; kaushik.nagendra@kw.ac.kr (N.K.K.); ehchoi@kw.ac.kr (E.H.C.)

**Keywords:** metabolic inhibitor, lung cancer, endoplasmic reticulum stress, mitochondrial activity

## Abstract

**Simple Summary:**

Metabolism targeting for cancer treatment is currently under research in an effort to classify molecules that may block major metabolic steps accompanying cancer development and malignant growth. The approach is to compromise or entirely inhibit the increased metabolic pathways in cancer cells by suppressing the enzymatic activity of the involved proteins. Targeting cancer metabolism unlocks the prospect of improving broadly appropriate drugs that can treat various cancer cell types and may facilitate an innovative class of anticancer molecules. Several analogs of metabolites are currently being tested as possible drug candidates for cancer metabolism. Determining the effect of these metabolites on lung cancer offers the potential for a new class of therapeutic agents for cancer treatment. Thus, the efficient use of metabolic inhibitors could be a clinically promising therapeutic scheme.

**Abstract:**

The pentose phosphate pathway (PPP) is the most common pathway in most cancer cells and stimulates antioxidant defense mechanisms and synthesis of biomolecule precursors. It is believed that cancer cells persistently ameliorate glucose flux into the PPP to maintain their anabolic requirements and adjust oxidative stress. TCGA analyses have indicated the upregulation of enzymes involved in PPP in lung cancer. Hence, the present study aimed to determine whether the pharmacological blockade of glucose 6-phosphate dehydrogenase (G6PD), the primary and rate-limiting enzyme involved in PPP, using 6-aminonicotinamide (6-AN), could induce antiproliferative activity in two lung cancer cell lines. Exposure to 6-AN suppressed lactate production and glucose consumption, modified the mitochondrial potential and redox balance, and thereby induced the endoplasmic reticulum (ER) stress to reduce lung cancer cell proliferation and govern cellular apoptosis. Collectively, this is the first study in which PPP blockade by 6-AN causes reactive oxygen species (ROS)-mediated apoptosis by ER stress in lung cancer cells. Further preclinical studies will be conducted to validate the biological applicability of these findings.

## 1. Introduction

Cancer cell development is continually supported by reprogrammed biological metabolism trials. To accommodate both anabolic and catabolic needs, rapidly proliferating cancer cells take up much higher levels of glucose compared with healthy normal cells. In addition to increased glucose uptake, aerobic glycolysis, also recognized as the Warburg effect, is a unique metabolic hallmark of cancer. In aerobic glycolysis, the metabolism of glucose to lactate is augmented by oxygen availability. It is worth mentioning that glycolysis is not a single pathway that requires glucose. The pentose phosphate pathway (PPP), which converts glycolysis, plays a vital role in cellular redox balance and nucleotide biosynthesis and is elevated in a wide variety of cancers [1]. The reshaped metabolic profile postulates growing cancer cells with prompt ATP production and carbon molecules for the composition of lipids, nucleotides, and proteins [2]. PPP is the foremost catabolic pathway of glucose by which tumor cells generate large quantities of ribose-5 phosphate, a predecessor of nucleotide formation, and NADPH, which is used to determine anabolic processes and to neutralize harmful reactive oxygen species (ROS) [3,4]. PPP consists of two functionally interrelated divisions: non-oxidative and oxidative branches [5]. Glucose-6-phosphate dehydrogenase (G6PD) functions in the first step of the oxidative branch to produce ribulose-5-phosphate (R5P) and NADPH, also known as a rate-limiting enzyme. On the other hand, the non-oxidative branch alters pentose sugars to intermediate glycolytic molecules such as fructose-6-phosphate and glyceraldehyde-3-phosphate through transketolase (TKT) and transaldolase (TALDO1). Additionally, several studies have reported that these molecules from oxidative (G6PD and 6-phospho-gluconate dehydrogenase) and non-oxidative (TKT-L1 and TALDO1) PPP routes that are frequently stimulated during carcinogenesis [3,6]. Lucarelli et al. claimed that the regulation of G6PD may vary with the accessibility of glucose: glucose funneling into the PPP oxidative branch directly mediates the redox homeostasis in renal cell carcinoma [7]; the study emphasized the consequences of PPP metabolism in the regulation of cancer cell survival and therapeutic challenges. The functional deactivation of rate-limiting enzymes of the PPP or the interference of glucose dispensing into G6PD-dependent reactions might serve as a promising approach to overcome acquired resistance to traditional existing therapies in solid cancers.

It is widely acknowledged that cancer cells are considered to have a high amount of ROS due to mitochondrial dysfunction and elevated levels of the NADPH-dependent oxidase complex (NOX) and resultant reducers, as a consequence of augmented glycolysis and the PPP cycle in contrast with normal healthy cells [8,9,10]. These intermediate reducers are quickly consumed in tumor cells to sustain accelerated anabolism, which is essential for their growth and survival [10,11]. A favorable scheme for obtaining preferential selectivity and therapeutic efficiency in cancer cells is to benefit from the basic difference in energy metabolism between normal and cancer cells. Targeting key molecules involved in biochemical metabolism activities in cancer cells could be a possible way to achieve therapeutic selectivity and feasibly counteract developed drug resistance and unwanted side effects [12,13].

The 6-aminonicotinamide (6-AN) inhibitor of PPP is in preclinical trials as a modulator of cytotoxicity of many antineoplastic drugs, predominantly DNA-damaging inducers [14]. Exceptionally, 6-AN is an effective inhibitor of G6PD, a PPP enzyme that is a critical step in NADPH synthesis and a ribose component prerequisite for biomolecule synthesis and DNA restoration [15,16]. Interestingly, Sharma et al. discovered that the simultaneous blockade of glycolysis and PPP by 2-deoxy-d-glucose (2-DG) and 6-AN, respectively, provoked oxidative stress that sensitized various aggressive cancer cell lines to radiation therapy via apoptosis, necrosis, and mitotic cell death modes [17]. Our group also showed that the use of the glycolytic inhibitor 2-DG improves the efficiency of cold plasma in blood and solid cancer cells to promote apoptosis [18,19].

In the current study, we examined the ability of 6-AN to induce apoptosis via endoplasmic reticulum (ER) stress, which results in ROS accumulation and leads to mitochondrial dysfunction in lung carcinoma cells.

## 2. Materials and Methods

### 2.1. Cell Culture and Reagents

A549 and H460 lung cancer cell lines were purchased from the Korean Cell Line Bank (KCLB, Seoul, Korea). A549 and H460 cells were grown in Dulbecco’s modified Eagle’s medium (DMEM, Welgene, Gyeongsan, Korea) and Roswell Park Memorial Institute (RPMI-1640, Welgene, Gyeongsan, Korea) medium, respectively, supplemented with 10% fetal bovine serum (RDTech USA), streptomycin (Welgene, Gyeongsan, Korea, 100 μg/mL), and penicillin (Welgene, Gyeongsan, Korea, 100 U/mL). To keep the cells in a healthy state, they were regularly passaged (every 2–3 days).

### 2.2. 6-AN Treatment

For 6-AN (Sigma-Aldrich, Seoul, Korea) treatment, cells were treated with various concentrations (1000, 500, 200, 100, 50, 20, 10, 5, or 1 μM). Cells were treated and incubated for 48 h before further experiments.

### 2.3. Cell Metabolic Activity and Clonogenicity Survival Assays

To determine cellular proliferation, A549 and H460 lung cancer cells were seeded at a density of 1 × 10^4^ in 96-well plates with a standard complete cell culture medium. After 48 h, the metabolic viability of the cells was tested using alamarBlue dye (Thermo Fisher Scientific, Waltham, MA, USA). Cellular viability was evaluated by measuring the fluorescence of alamarBlue conversion, as previously described [20,21,22]. For the clonogenicity assay, A549 and H460 cells were seeded in 60-mm cell culture dishes in triplicate at cell concentrations expected to produce 25–90 colonies/dish. After 48 h of incubation, both cells were treated with low (10 μM) and high (200 μM) 6-AN concentrations. Cells were fixed and stained with crystal violet after being cultured for the next 12–16 days, and colonies were manually counted using a cell counter.

### 2.4. Metabolic Energy Marker Analysis

Glucose consumption, lactate level, and intracellular ATP levels were detected using a commercially available Glucose Assay Kit (Sigma-Aldrich, Seoul, Korea), the EnzyChrom™ Lactate Assay Kit (Bioassay Systems, Hayward, CA, USA), and EnzyLight^TM^ ATP assay kit (Bioassays Systems, Hayward, CA, USA), respectively [18].

### 2.5. Flow Cytometry Analysis

Flow cytometry analysis of mitochondrial membrane potential (MMP) was performed using Mitoflow reagent (Cell technology, Fremont, CA, USA); all procedures were performed according to the manufacturer’s instructions. Briefly, both control and treated lung cancer cells were harvested and stained with mitoflow reagent as protocol. Afterwards, samples were immediately interpreted with the BD FACSVerse system (BD Biosciences, Seoul, Korea) using the FACS suite software.

### 2.6. Live/Dead Cell Staining

The control and 6-AN treated cells were stained with the Live/Dead viability kit (Molecular Probes, Invitrogen, Eugene, OR, USA) to check viability imaging at 48 h time points. Briefly, these cells were post-incubated with the live/dead staining solution consisting of 0.05% of 4 mM Calcein-AM and 0.2% of 2 mM ethidium homodimer-1 for 40 min at room temperature and were then photographed using a confocal fluorescence microscope (Olympus, Seoul, Korea).

### 2.7. qRT-PCR

Total RNA from control and 6-AN treated cells was isolated manually using Tri reagent (Ambion, Naugatuck, CT, USA). To synthesize complementary DNA template, 2 µg RNA was spent using MMLV reverse transcriptase (Enzymonics, Daejeon, Korea) superMix consisting of 30 mM MgCl_2_, reaction buffer (500 mM Tris-HCl, 750 mM KCl, and 100 mM dithiothreitol), deoxynucleotide triphosphates (dNTPs), and RNase inhibitor, according to the manufacturer’s instructions. The mRNA expression levels of genes were studied by qRT-PCR on an iCycler IQ Real-Time Detection System (Bio-Rad, Hercules, CA, USA). The results are shown as fold change normalized to the control. All primers were designed and purchased from Macrogen (Seoul, Korea; Table 1).

### 2.8. Intracellular ROS Determination (H2DCFDA)

The levels of intracellular reactive species were measured using the cell-permeant 2′,7′-dichlorodihydrofluorescein diacetate (H2DCFDA, Invitrogen) as described in our previous report [19]. Initially, control and treated lung cancer cells were harvested at the desired time points. Then, cells were washed and incubated with ROS indicator H2DCFDA for 30–40 min at room temperature, followed by flow cytometry analysis using the FACS suite software.

### 2.9. Evaluation of the NADP/NADPH Ratio

The ratio of cytoplasmic NADP/NADPH was calculated using a NADP/NADPH quantification assay kit (Sigma-Aldrich, Seoul, Korea) according to the manufacturer’s instructions. Control and treated cells were collected after 48 h, and NADP and NADPH levels were measured.

### 2.10. Ki67 Immunofluorescence

Cells were fixed with 4% paraformaldehyde, permeabilized, and incubated with rabbit monoclonal Ki-67 antibodies (Cell Signaling, 1: 200), in phosphate buffered saline (PBS) consisting of 1% BSA and 0.2% Triton X-100 at 4 °C overnight. Nuclei were visualized with 4,6-diamidino-2-phenylindole (DAPI; Sigma-Aldrich, Seoul, Korea) and cells were photographed at 40× with a confocal fluorescence microscope (Olympus, Seoul, Korea).

### 2.11. Apoptosis Assays

Apoptotic or programmed cell death is defined by cellular shrinkage, nuclear fragmentation, blebbing of the plasma membrane, and eventual apoptotic body formation [23]. For apoptotic cell death detection, an Annexin V-FITC Apoptosis Detection Kit I (Pharmingen, San Diego, CA, USA) was used for control and treated cells at 48 h post-incubation and investigated by flow cytometry. A total of 10,000 cells/events were considered using the FACS suite software. The nuclei were counterstained with DAPI and photographed using a confocal microscope.

### 2.12. TCGA Data Analysis

For transcriptome sequencing information, TCGA lung adenocarcinomas (566 patient samples) were accessed from cBioPortal for cancer genomics (https://www.cbioportal.org/, accessed on 6 August 2021). Gene expression levels were determined by RNA-Seq using the RSEM values. 

### 2.13. Statistical Analysis

All results are shown as the mean + standard deviation (SD) from three independent sets of experiments. Statistical analyses were performed using the parametric Student’s t-tests (unpaired, two-tailed) in the GraphPad Prism 9.2 software. Significance was designated as * *p* < 0.05, ** *p* < 0.01, *** *p* < 0.001 vs. controls.

## 3. Results

### 3.1. PPP Inhibitor 6-AN Induces Apoptosis in Non-Small Lung Cancer Cells

**Figure 1:**
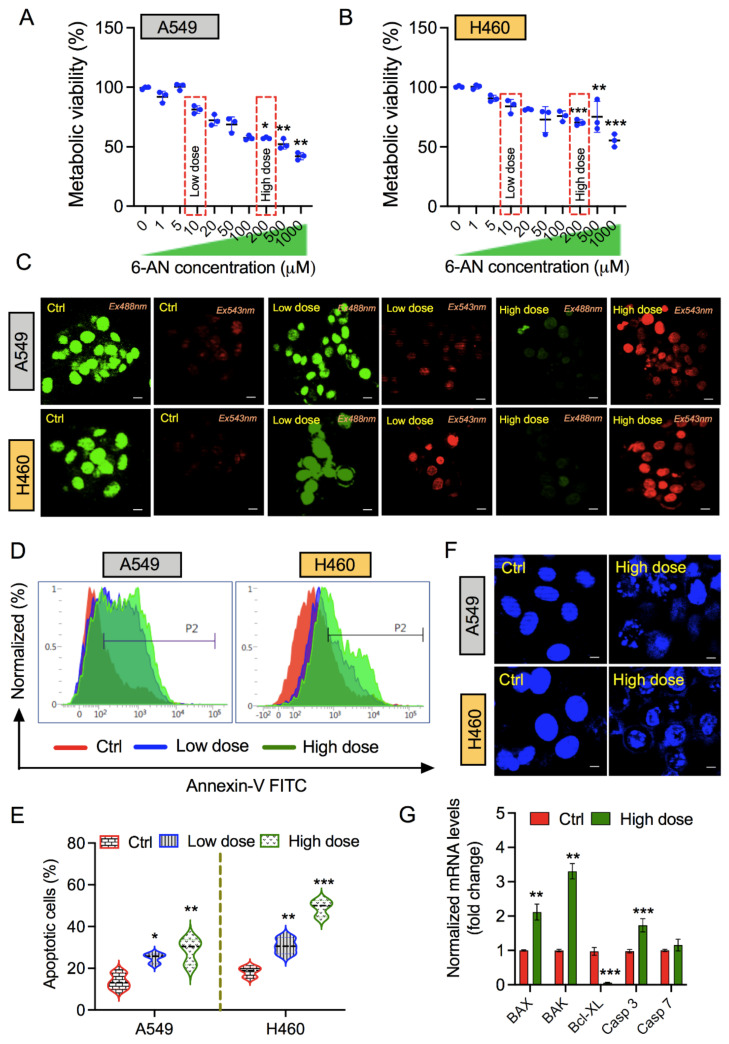
**Pentose phosphate pathway (PPP) inhibitor 6-aminonicotinamide (6-AN) promotes apoptotic cell death in non-small lung cancer cells**. (**A**,**B**) Metabolic activity of A549 and H460 lung cancer cells, respectively, when treated at 1000, 500, 200, 100, 50, 20, 10, 5, or 1 μM concentrations of 6-AN. (**C**) Live/dead cell viability assay of A549 and H460 cells treated for 48 h post 6-AN treatment. (**D**) Annexin V-FITC flow cytometric analysis of apoptosis in A549 and H460 lung cancer cells, treated at low (10 μM) and high (200 μM) doses of 6-AN treatment. (**E**) Representative graph of total apoptotic percent population of both cell lines observed at indicated doses. (**F**) Immunofluorescence nuclear 4,6-diamidino-2-phenylindole (DAPI) stained images of A549 and H460 cells treated at high doses of 6-AN. (**G**) qPCR analysis of apoptosis related markers in H460 cells in presence or absence of 6-AN treatment. * *p* < 0.05; ** *p* < 0.01; *** *p* < 0.001.

In the present study, we first examined the ability of 6-AN to induce cytotoxicity in lung cancer cells. A549 and H460 lung cancer cells were treated with 6-AN at various concentrations (1–1000 µM) for the next 48 h post-treatment. As shown in Figure 1A,B, inhibition of PPP using 6-AN suppressed the metabolic activity of both lung cancer cell lines in a dose-dependent manner. To confirm this, we stained A549 and H460 cancer cells for live/dead cell imaging after 6-AN treatment. Interestingly, there was a dramatic reduction in the live (green) cell population at a low dose (10 µM) and high dose (200 µM) 6-AN treatment in both cell lines, as observed by confocal microscopy. In addition, an increase in the dead cell population was also observed in the treated lung cancer cells under the indicated treatment conditions (Figure 1C).

Next, to determine whether 6-AN treatment can promote programmed cell death (i.e., apoptosis of lung cancer cells), we tested A549 and H460 cancer cells for Annexin-V staining following treatment at low and high treatment conditions. Annexin-V is a phospholipid-binding protein with a high affinity for phosphatidylserine, which is frequently used as a sensitive probe for apoptosis detection. During early apoptosis, membrane alterations occur where translocation of phosphatidylserine occurs from the inner side of the plasma membrane to the extracellular layer. Flow cytometry analysis indicated a subsequent increase in the number of apoptotic cell populations in A549 and H460 cancer cells after 6-AN treatment; however, this effect was higher in H460 cells than in A549 cancer cells (Figure 1D,E). Moreover, DAPI staining showed damaged cellular morphology in both cell types, as seen by immunofluorescence microscopy (Figure 1F). Next, we checked the expression of apoptosis-related genes such as BAX, BAK, Bcl-XL, Casp3, and Casp 7 in 6-AN-treated cells. Our results show that 6-AN increased the expression of pro-apoptotic genes (*BAX*, *BAK*, *Casp 3*, and *Casp 7*), but reduced the expression of anti-apoptotic *Bcl-XL* gene expression (Figure 1G). Taken together, these results indicate the potential of 6-AN for cytotoxicity in lung cancer cells.

### 3.2. Exposure of 6-AN Reduces Clonogenicity of Lung Cancer Cells

**Figure 2:**
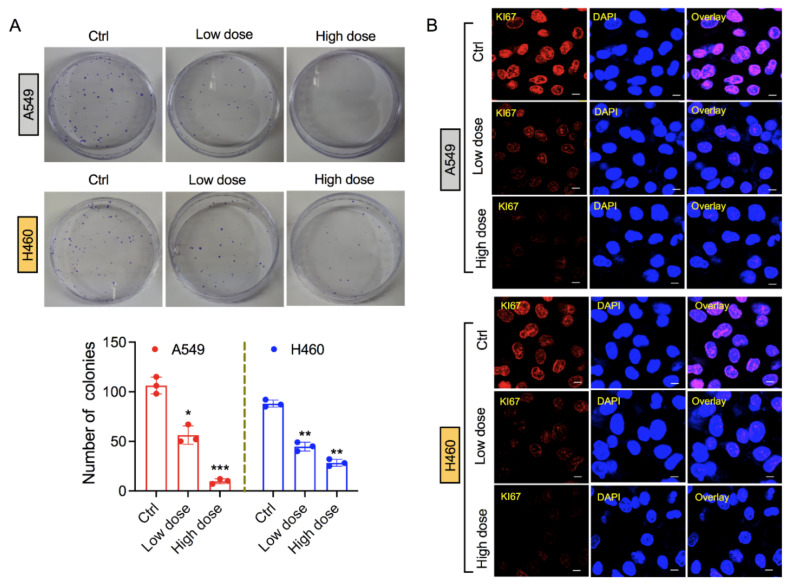
**Exposure of6-aminonicotinamide (6-AN) suppresses the proliferation of lung cancer cells.** (**A**) Representative images of colony-forming assays in A549 (upper panel) and H460 (lower panel) stained with crystal violet after indicated doses of 6-AN treatment for 48 h. Graphs indicate the number of colonies formed after each treatment condition in both cell lines. (**B**) Cellular expression of the Ki-67 proliferation marker in A549 and H460 lung cancer cells after 48 h of 6-AN treatment at low (10 μM) and high (200 μM) doses. Cells were labeled by immunofluorescence for Ki-67 (red) with 4,6-diamidino-2-phenylindole (DAPI; blue) as the nuclear counterstain. * *p* < 0.05; ** *p* < 0.01; *** *p* < 0.001.

After confirming the reduction in cellular metabolic viability of lung cancer cells by 6-AN treatment, we next investigated the survival of treated lung cancer cells following 6-AN post-incubation. The 6-AN decreased colony formation in both A549 and H460 cancer cell lines at low and high doses (Figure 2A). In addition, we tested the antiproliferative activity of 6-AN in lung cancer cells by measuring the expression of Ki-67, a proliferation marker that is generally highly expressed in growing cells and weakly expressed in quiescent cells [24]. In our experiments, we observed a decline in Ki-67 fluorescence intensity after 6-AN treatment in both A549 and H460 cancer cells, as seen by confocal microscopy (Figure 2B). This result showed that 6-AN was capable of reducing the proliferation of lung cancer cells.

### 3.3. Exposure of 6-AN Alters the Metabolic Parameters of Lung Cancer Cells

**Figure 3:**
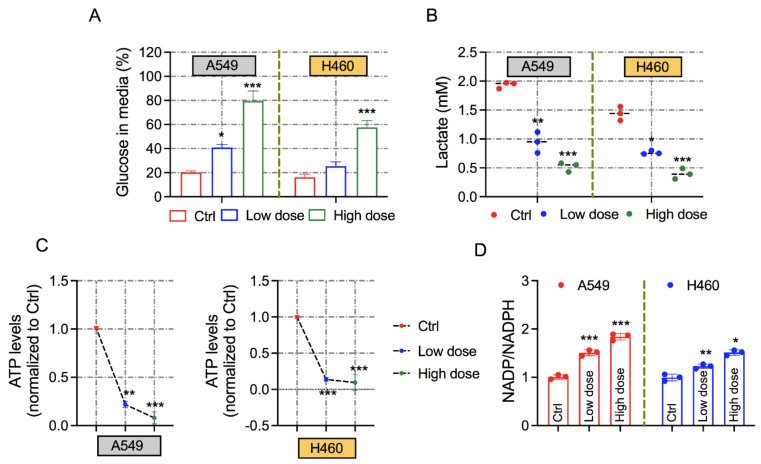
**Treatment with 6-AN modulates metabolic indicators of lung cancer cells.** (**A**) Glucose consumption was determined at low (10 μM) and high (200 μM) concentrations of 6-AN in A549 and H460 lung cancer cells. (**B**) Graph of lactate levels in A549 and H460 cancer cells under both conditions. (**C**) Measurement of cellular ATP levels in A549 and H460 cancer cells following treatment with low (10 μM) and high (200 μM) 6-AN treatments. (**D**) Analysis of the NADP+/NADPH ratio in A549 and H460 cancer cells after 48 h of 6-AN treatment at low and high doses. * *p* < 0.05; ** *p* < 0.01; *** *p* < 0.001.

To identify the mechanisms that are essential for cytotoxic and antiproliferative effects, we examined the effects on cancer metabolic reprogramming following inhibition of PPP via 6-AN treatment. In this regard, we questioned whether 6-AN disturbs glucose intake and lactate production, which are predominantly exacerbated in tumor cells. We observed that the glucose concentration in the extracellular medium was higher after 6-AN treatment at both low and high doses in both cancer cell lines, indicating less consumption compared with control cells (Figure 3A). In addition, PPP inhibition by the treatment of 6-AN simultaneously decreased the concentration of lactate in the cellular supernatants of A549 and H460 cancer cells (Figure 3B). Furthermore, intracellular ATP levels were notably reduced in 6-AN-treated A549 and H460 cancer cells following low-and high-dose treatment conditions (Figure 3C). In PPP metabolism, NADPH is the primary source of the oxidative branch, which nourishes the pool of reduced glutathione to stabilize the redox state. As expected, 6-AN treatment significantly enhanced the NADP/NADPH ratio in the A549 and H460 lung cancer cells (Figure 3D). Collectively, these findings demonstrate that 6-AN treatment ameliorates mitochondrial energy metabolism in cancer cells.

### 3.4. 6-AN Increases Intracellular ROS and ER Stress, and Affects Mitochondrial Membrane Potential

**Figure 4:**
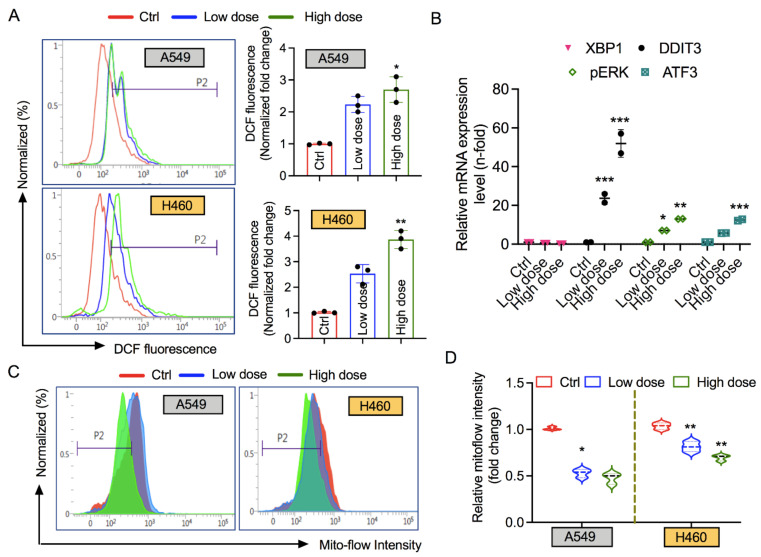
**Treatment with 6-aminonicotinamide (6-AN) enhances oxidative and endoplasmic reticulum (ER) stress, and affects mitochondrial membrane potential (MMP)**. (**A**) Intracellular reactive oxygen species (ROS) levels were determined in both A540 and H460 cancer cells treated with low (10 μM) and high (200 μM) concentrations of 6-AN treatments using flow cytometry. Representative graphs indicate the intensity of DCF fluorescence in 6-AN treated cancer cells normalized to the control. (**B**) qRT-PCR analysis of mRNA expression of ER stress-related genes such as XBP-1, DDIT3, pERK, and ATF3 following 6-AN treatment in H460 cancer cells under both conditions. (**C**) Mitochondrial depolarization levels in A549 and H460 cancer cells following low (10 μM) and high (200 μM) concentrations of 6-AN treatments, detected by flow cytometry with the Mitoflow reagent. (**D**) Representative graphs of levels of Mitoflow intensity in A549 and H460 cancer cells, calculated from panel C as per the indicated treatment conditions. * *p* < 0.05; ** *p* < 0.01; *** *p* < 0.001.

In PPP, the generated NADPH can be exploited for ROS detoxification or lipid biosynthesis [25]. We inspected the intracellular ROS content in A549 and H460 lung cancer cells following 6-AN treatment. Strong DCF fluorescence staining was detected in both cancer cells treated with 6-AN; however, this effect was more prominent in the high-dose treatment in both cell lines (Figure 4A). These data suggest that the apoptotic cytotoxicity induced by 6-AN via PPP inhibition could be partly mediated by the ROS mechanism. A number of studies have suggested that several stressful conditions such as nutrient deprivation and hypoxia can act as growth-limiting factors for cancer cells, resulting in the activation of unfolded protein response (UPR) signaling. Under normal conditions, nutrient starvation [26,27] or excess nutrients in cancer cells generate ER stress [28,29]. Based on this knowledge, we carried out real-time qPCR analyses of UPR genes that were liable for ER stress activation. The mRNA levels of *DDIT3*, *pERK*, and *ATF3* were highly upregulated upon 6-AN treatment in H460 cancer cells; however, *XBP1* was not (Figure 4B). As cellular mitochondria are a key source of intracellular oxidants, we hypothesized that mitochondrial depolarization could be linked to lung cancer cell death following 6-AN treatment. Therefore, we tested the mitochondrial membrane potential (MMP) after 6-AN treatment. Remarkably, flow cytometry analysis showed that 6-AN treatment tended to trigger an early depolarized mitochondrial state in A549 and H460 cancer cells at high and low doses, as seen by a reduction in Mitoflow intensity (Figure 4C,D). These data suggest that inhibition of PPP by 6-AN could induce ER stress in lung cancer cells, which might contribute to the pathway controlling apoptotic cancer cell death.

### 3.5. 6-AN Modulates the Expression of Key Enzymes Involved in PPP

**Figure 5:**
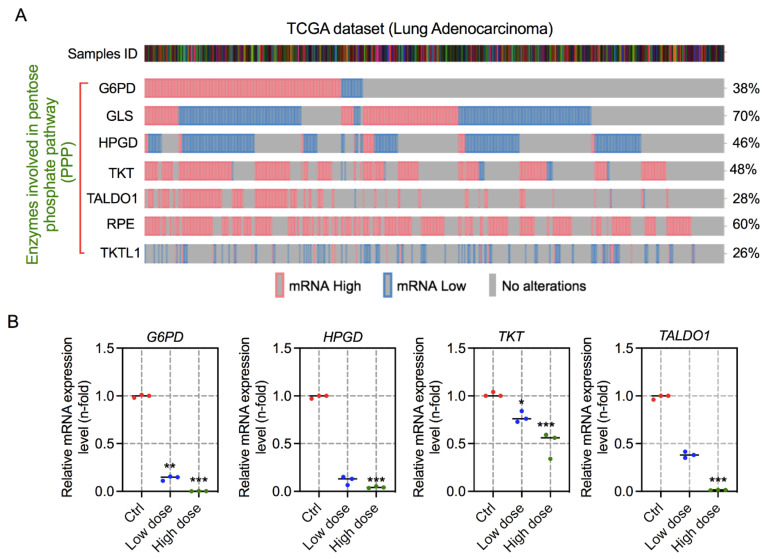
**Treatment with 6-aminonicotinamide (6-AN) reduces the expression of the main enzymes involved in the pentose phosphatepathway (PPP)**. (**A**) TCGA analysis of the main enzymes involved in PPP for mRNA expression. (**B**) qRT-PCR analysis of mRNA expression of PPP-related genes such as G6PD, HPGD, TKT, and TALDO1 following 6-AN treatment in H460 cancer cells under both conditions. * *p* < 0.05; ** *p* < 0.01; *** *p* < 0.001.

Both the non-oxidative and oxidative branches of the PPP take place in the cytosol, where several enzymes are involved in metabolism regulation [30]. To determine the expression of key enzymes in lung adenocarcinoma, TCGA data analysis was performed. These analyses demonstrated that mRNA expression of G6PD, glutaminase (GLS), hydroxy-prostaglandin dehydrogenase 15-(NAD) [HPGD], TKT, TALDO1, ribulose-5-phosphate-3-epimerase (RPE), and transketolase-like-1 (TKTL1) were more abundant in lung cancer patient samples (Figure 5A). To substantiate our results by 6-AN treatment in lung cancer cells, we checked the expression of PPP-related genes such as G6PD, HPGD, TKT, and TALDO1 in these cells 48 h after 6-AN treatment. Notably, 6-AN noticeably inhibited the induction of the tested genes in H460 cancer cells. Interestingly, the expressions of G6PD, HPGD, and TALDO1 were reduced to a greater extent in both doses, as shown in Figure 5B.

## 4. Discussion

Consistent with earlier works [31,32,33], our findings support the idea that bioenergetic modulation is an emerging strategy to overcome cancer. Kaushik et al. (2015), and other research groups, have presented interesting results regarding the use of a synergistic combination of 2-DG with cold plasma on blood cancer cells [18], and with metformin in several tumor cells [34]. Notably, these studies have proven that these cells are highly sensitive to glycolysis inhibition by 2-DG. Therapies that trigger tumor cellular metabolism are supposed to abolish tumor development or re-sensitize tumor cells to radio/chemotherapy. At present, several anti-metabolites and pharmacological inhibitors targeting metabolic pathway enzymes are being considered in preclinical animal models or clinical phase trials [35]. PPP is demanding for cancer treatment and prevention owing to the formation of NADPH and R5P, which play key roles in regulating metabolism, DNA damage response, and proliferation in tumor cells. In cancer cells, the non-oxidative branch of PPP predominantly engages in de novo nucleotide synthesis [36]. Our findings are in agreement with earlier studies that verified that the expression of oxidative and non-oxidative PPP-related enzymes is upregulated in lung cancer using TCGA analysis. The downregulation of these enzymes was observed to be caused by the inhibition of PPP via 6-AN in lung cancer cells (Figure 5). The increased enzymatic expression of G6PD, a central enzyme of the oxidative branch of the PPP, has been reported in a wide variety of cancer types [37]. In this study, the effect of 6-AN, a competitive G6PD inhibitor, was explored in lung cancer cells. Previously, 6-AN was also used in chemotherapy to treat diverse malignancies [38]. PPP is the leading fate of intracellular glucose consumption after glycolysis, which supplies the necessity for anabolic precursors [1,39]. Another characteristic that should be taken into consideration is the fact that inhibition of G6PD causes a rise in its substrate G6P, which eventually inhibits hexokinase enzymatic activity, a major enzyme in the glycolysis pathway. This conclusion could explain the decline in glucose consumption and lactate production following 6-AN treatment in lung carcinoma cells (Figure 3A,B). 

Ki67 immunostaining and colony formation assays reveal that 6-AN suppresses the proliferation index in A549 and H460 cancer cells (Figure 2). Additionally, 6-AN treated lung cancer cells displayed the highest Annexin-V positivity, in agreement with the increase in apoptotic cell death levels (Figure 1D,E). We also anticipated that the inhibition of PPP may cause ROS-induced apoptosis in lung cancer cells. Stimulation of cellular ROS generation initiated by either oxidative stress or blockade of NADPH-generating pathways might lead to the expansion of the cellular NADP+/NADPH ratio. As expected, inhibition of PPP upon 6-AN treatment decreased NADPH availability (Figure 3D) and increased ROS content (Figure 4A). Furthermore, the accumulation of ROS could increase ER stress and aggravate ER stress-induced cell death [32]. ER stress can be induced by energy fluctuations and nutrient deprivation. These perturbations produce a gathering of misfolded and unfolded proteins in the ER, which triggers a mechanical signal transduction pathway called the unfolded protein response (UPR) [40]. UPR signaling in cancer is a contradiction, with both pro-apoptotic and pro-survival pathways are concurrently upregulated; however, the balance is angled toward malignancy of cancer cells. It is believed that ER stress participates in apoptosis when extracellular stress is chronic or too harsh [41]. We observed that DDIT3, pERK, and ATF3 mRNA levels were mainly upregulated upon 6-AN treatment in lung cancer cells (Figure 4B). Chemotherapeutic drugs that stimulate apoptosis in cancer cells are associated with rapid collapse of mitochondrial membrane potential [42]. Mitochondrial stress was also observed in 6-AN-treated lung cancer cells, as evidenced by decreased ATP levels, increased mitochondrial depolarization, and ROS production (Figure 3 and Figure 4). These findings suggest that fluctuating cellular energy metabolic pathways, such as glucose flux, or genetic modification of signaling pathways, could extensively affect PPP.

## 5. Conclusions

Pharmacological inhibition of PPP leads to a reduction in the proliferation of lung cancer cells, likewise targeting the PPP enhanced cellular oxidative stress and induced ER stress in these cells through 6-AN treatment. Our results suggest that the inhibition of PPP by 6-AN can function as a prospective targeted therapeutic opportunity in lung cancer cells. Since PPP serves as a major source of NADPH production, it could be an ideal target for controlling redox homeostasis in metabolic disorders, including cancers. G6PD and 6PGD are the major enzymes involved in PPP, which catalyze the reactions to generate NADPH; therefore, blocking the function of these enzymes would eventually restrict DNA replication and damage repair responses. Hence, PPP enzymes such as G6PD, including 6PGD and TKT, are promising targets for the treatment and anticipation of metabolic diseases.

## Figures and Tables

**Table 1:** List of primer sequences used in this study.

Gene Name	Sequence (5′-3′)
*ACTIN*-forward	GGC ATC CTC ACC CTG AAG TA
*ACTIN*-reverse	AGG TGT GGT GCC AGA TTT TC
*G6PD*-forward	GAAACGGTCGTACACTTCGG
*G6PD*-reverse	CCGATGCACCCATGATGATG
*TKT*-forward	GTGCCTCTAAGACACCCTGT
*TKT*-reverse	GTGAAAGGGGAGCTGAGAGT
*HPGD*-forward	TAGCGCTGGTGGATTGGAAT
*HPGD*-reverse	GACCAAAATGTCCAGTCTTCCA
*TALDO1*-forward	TAAAGAAGATTCCGGGCCGA
*TALDO1*-reverse	TCCCAGGTTGATGACAGCTT
*XBP1*-forward	GGAGTTAAGACAGCGCTTGG
*XBP1*-reverse	CACTGGCCTCACTTCATTCC
*DDIT3*-forward	CAGAGCTGGAACCTGAGGAG
*DDIT3*-reverse	TGTTTATGGCTGCTTTGGTG
*pERK*-forward	CCAGCCTTAGCAAACCAGAG
*pERK*-reverse	TCTTGGTCCCACTGGAAGAG
*ATF3*-forward	TTTGCCATCCAGAACAAGC
*ATF3*-reverse	CATCTTCTTCAGGGGCTACCT

G6PD: Glucose-6-phosphate dehydrogenase; TKT, transketolase; HPGD, hydroxy-prostaglandin dehydrogenase 15-(NAD); TALDO1, transaldolase; XBP1, X-box binding protein 1; DDIT3, DNA damage-inducible transcript 3; pERK, phospho-extracellular signal-regulated kinases; ATF3, activating transcription factor 3.

## Data Availability

All data are included in this manuscript.
